# Epileptic seizure detection using CHB-MIT dataset: The overlooked perspectives

**DOI:** 10.1098/rsos.230601

**Published:** 2024-05-29

**Authors:** Emran Ali, Maia Angelova, Chandan Karmakar

**Affiliations:** ^1^ School of Information Technology, Deakin University, Melbourne Burwood Campus, Melbourne, Victoria 3125, Australia; ^2^ Aston Digital Futures Institute, EPS, Aston University, Birmingham, UK; ^3^ Institute of Biophysics and Biomedical Engineering, Bulgarian Academy of Sciences, Sofia, Bulgaria

**Keywords:** epilepsy, seizure, machine learning, seizure event detection, cross-subject analysis, health informatics

## Abstract

Epilepsy is a life-threatening neurological condition. Manual detection of epileptic seizures (ES) is laborious and burdensome. Machine learning techniques applied to electroencephalography (EEG) signals are widely used for automatic seizure detection. Some key factors are worth considering for the real-world applicability of such systems: (i) continuous EEG data typically has a higher class imbalance; (ii) higher variability across subjects is present in physiological signals such as EEG; and (iii) seizure event detection is more practical than random segment detection. Most prior studies failed to address these crucial factors altogether for seizure detection. In this study, we intend to investigate a generalized cross-subject seizure event detection system using the continuous EEG signals from the CHB-MIT dataset that considers all these overlooked aspects. A 5-second non-overlapping window is used to extract 92 features from 22 EEG channels; however, the most significant 32 features from each channel are used in experimentation. Seizure classification is done using a Random Forest (RF) classifier for segment detection, followed by a post-processing method used for event detection. Adopting all the above-mentioned essential aspects, the proposed event detection system achieved 72.63% and 75.34% sensitivity for subject-wise 5-fold and leave-one-out analyses, respectively. This study presents the real-world scenario for ES event detectors and furthers the understanding of such detection systems.

## 1. Introduction

Epilepsy is one of the most prevalent cranial conditions in human beings that affects millions of people around the world [1]. It brings down the quality of life for those affected, regardless of age, race or geographic location. A transitory incidence of signs or symptoms associated with abnormally excessive and synchronized neuronal activity in the brain is known as an epileptic seizure (ES) or generally a seizure [2,3]. Although 75% of epilepsy cannot be prevented [4], with adequate medical treatment and diagnosis, many epilepsy patients can be cured [5–7]. An appropriate diagnosis is required to identify the seizure’s type, nature and location for effective treatment of ES. Manual detection of ES from very long and continuous data is distressful and infeasible. Thus, an automated ES detection system is used independently or as a clinical support system. Various diagnostic options are available for identifying ES occurrence, including blood tests, physiological examination and different signal analysis processes. The signal modalities used for ES detection are accelerometry (ACM) [8–10], ECG [11–13], EEG [14–18], MRI [19,20] and so forth. Continuous electroencephalography (cEEG) is considered the gold standard for seizure detection among all available modalities [15,16,21]. The scalp electroencephalography (scalp EEG) signal is preferred over intracranial EEG for diagnostic and general purposes as being a non-invasive process [22,23]. One of the most extensive continuous scalp EEG datasets is the CHB-MIT dataset, and numerous studies have been conducted using this dataset [24].

Diagnosis of ES using EEG is simply classifying or separating the seizure portion of the EEG signal from the normal (non-seizure) one. This classification can be done using threshold-, machine learning (ML)- or deep learning (DL)-based methods. Among these methods, ML methods are preferred concerning the model’s generalizability, feature explainability and detection latency [25,26]. They use different explainable (human perceivable) features that define the seizure event and differentiate from the normal signal [27–31]. In medical diagnosis, only classifying seizure from non-seizure data is not enough; rather, a proper explanation of the classification process is required. The explanations include which features are responsible for separating the seizure data from non-seizure, and based on what physical characteristics those features can do so. These explanations help medical experts map the feature with corresponding biological or chemical factors for proper treatment planning.

One of the best ways to get the actual scenario for seizure detection using data-driven methods is to test the method’s generalizability power. To do so it is required to train the model with sufficient data and test the trained model using the rest of the unseen data. However, continuous EEG data is typically imbalanced and has fewer (seizure) events than the normal signals. Most of the studies found in the literature that used large public datasets (especially the CHB-MIT dataset) have not considered all the data for testing the performance of their method. These types of experiments on partial data may temporarily show significantly higher performance. Still, they will struggle to perform well once they encounter the actual scenario (the imbalanced nature of continuous data). All the studies that considered different datasets such as the Bonn dataset considered all the data for observation [32–38]. However, it is a minimal dataset, too small to test the generalizability of a model. Other studies that used private data showed the performance scores on the entire dataset, but due to availability issues, they cannot be tested [14,39,40]. Most of the studies that were done on the CHB-MIT dataset considered only the records that have seizures in them [41–43]. The majority of the previous studies discarded the records with no seizure events in them, even though those records are part of the continuous EEG signal. Some studies even considered a portion of the data or excluded some based on their choice for analysis [44–48]. When the data are balanced for testing or portions of data are left out of consideration in the analytical process, this can greatly impact the performance score. Different data alteration processes are often used in many studies, such as filtering, augmentation, scaling and related preprocessing [49–52]. This type of alteration may change the original property of the signal [53]. Data in its entirety is demanded and also legitimately evaluated to test a model’s capability.

Similar to any other physiological signals, EEG signals are highly nonlinear and non-stationary [54]. The inter-subject variability in EEG signal is much higher than the intra-subject changes [55,56]. Thus, the random choice of the signal data to be included in training and test sets for classification (for random fold-based splitting) may lead to a data leakage problem. In this case, the data segments from the same subjects may appear in both the training and test sets. This situation gives the model a better idea about the data pattern available in the test set before testing, and it might produce better results that are not realistic. This data leakage situation leads to the need for cross-subject analysis so that data leakage can be prevented [51,57–60]. Most of the previous studies ignored this factor and did not consider cross-subject analysis [18,42,61–63]. Some subject-specific or patient-specific systems were investigated in earlier studies, where the training and test were done on the same-specific subject [24,57,64–66]. However, this approach has a critical pitfall: the system needs to be trained on the subject before it can be used for that same subject. Additionally, a large amount of data collected from other patients cannot be used in this system design. Thus, it hinders the system from being robust and generalized for all the patients.

Different approaches can be used to detect the seizure occurrence from the EEG signal. The most common approach for ES classification methods is segment classification [37,67,68]. This approach divides the continuous raw EEG signals into small chunks called segments. This process of dividing the signal into small pieces is called segmentation. The long seizure events are also divided and distributed into several segments during segmentation. The ML model is trained on some (e.g. 70–80% of total segments) randomly selected segments. Then, the trained model is tested on separate random segments (e.g. 30–20% of total segments) that have not been presented to the model during training. The performance of the classifier model is evaluated based on how many test segments are correctly identified or not [27,37,59,60,63,68–70]. Only considering the segment detection does not always guarantee that the correctly detected segments are from the same event. So, the question remains: is the model able to detect seizure events correctly rather than just detecting random segments? The other approach to classification is event classification. In this approach, the event with its original length can be separated and detected. The entire length of seizure or non-seizure can be separated and used for analysis to classify seizure or non-seizure. Another alternative to the entire event separation for event detection is to combine segment results. The detected segments can be sorted in their original sequence to check if all the or majority of the segments in the entire event are being detected correctly. Event detection is a more realistic consideration for real-world application [41,42,61,62] because it specifies if a model is capable of detecting seizure events, not just random segments. Unfortunately, most of the previous studies did not focus on this important factor but rather focussed only on random seizure segment detection. Several previous studies worked on event detection that includes entire event separation and detection (as mentioned above) and seizure onset (the initial portion(s) of the seizure event) detection [41–43]. Only one of these studies tackled data leakage and random segment detection problems by observing the event detection for the cross-subject setup [41].

All the factors mentioned above are associated with the real-world situation of a seizure detection system. These must be noticed when developing an actual ES detection system. Although some studies in the literature considered one or two factors, almost all of them lack considering all these factors altogether. To address all the unnoticed issues, in this study, we investigated the detection of ES events using multiple channels of continuous EEG signal from the most commonly used and publicly available scalp EEG dataset, the CHB-MIT dataset. However, to detect the seizure events using the ML model, the cross-subject classification strategy was employed and considered all the data from the dataset unchanged during testing. The main aim of this study is to investigate the efficacy of the cross-subject seizure event detection system using the ML model on all of the original data from the CHB-MIT dataset, which reveals the actual seizure detection scenario. To the best of our knowledge, this is one of the first studies that considers all the above-mentioned factors for ES detection. Below are a number of objectives that support this study’s main aim, which are the main novel contributions of our study:

— Detection of ES using ensemble ML models with explainable features.
— Observing the model’s efficacy using the real-world data imbalance scenario. To do that, the unchanged data from the entire dataset is used for testing.
— Observing the cross-subject (subject-wise, SW) analysis of ES to ensure the inter-subject variability is intact and there is no data leakage between the training and test sets.
— Implementing the original goal of a seizure detection system, that is, to detect ES events instead of detecting random seizure segments.
— Combining all of these criteria to observe the efficacy of a seizure detection system.

The rest of the article is organized by starting with the data and methodological description in §2. It includes the dataset description, feature extraction and selection process. This section describes the classification method (model), parameters used in the classification, post-processing method and performance metrics. The obtained result is described in §3, and the comparison with the most relevant articles with detailed discussion is presented in §4. Finally, the report is concluded with the findings in §6 followed by the limitations of this study and the future direction of this study in §5.

## 2. Material and methods

### 2.1. Methodology

**Figure 1.**
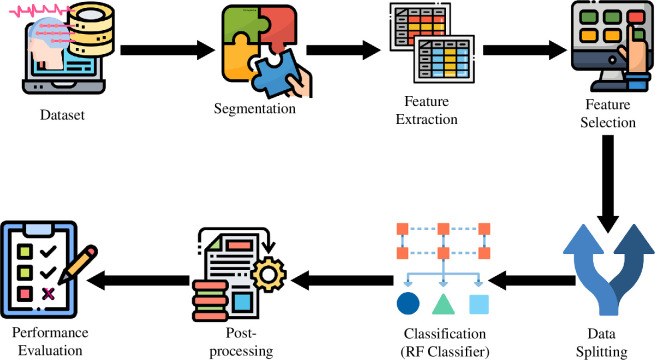
Block diagram of the event detection system demonstrating the steps followed for this study. Starting with raw EEG signal data collection from the dataset, the raw signal is cut into segments and different features are extracted from each segment of each channel. Feature selection is the next to select important features only. After properly splitting in the appropriate ratio for training and testing, data are sent to the RF classifier to be classified as seizure or non-seizure. Post-processing for event detection is done with the classified results and evaluated with proper performance measurement metrics.

The methodology that we followed is described using the block diagram presented in figure 1. It includes data collection, segmentation and feature extraction, feature selection, data splitting and model training, testing and result generation, result post-processing and performance evaluation. The appropriate data from the CHB-MIT dataset are considered for the analyses of this study. The data are divided channel-wise and cut into segments for each of the channels. Different features are extracted from each segment of the raw EEG signal. Feature selection is applied to select more meaningful features for seizure detection. After properly splitting the data SW into appropriate ratios for training and testing, they are sent to the random forest (RF) classifier to be classified as seizure or non-seizure. Data splitting and classification that includes training, validation and testing of the RF model are repeated for *k* times SW, where *k* = 5 is for 5-fold analysis and *k* = 24 is for leave-one-out (LOO) analysis. Although the LOO analysis is our main target, we expanded the experiment to 5-fold to include more variations in the test set and to train the model with less data to observe the performance variations. To investigate the results in more detail, the segment-based results are also explored and compared before proceeding with event detection. This will also facilitate the comparison of the effect of subjective data leakage, which is one of the factors we considered in this study. Post-processing for event detection is done from the classified results and evaluated with proper metrics. Each of these steps is described in detail in the following sections. The entire analytical process (all the steps described) is done with the most popular programming language, Python. The flowchart in the electronic supplementary material presents a more detailed view of how the analysis was done using different steps in figure 1 of appendix B.

### 2.2. Dataset

The CHB-MIT scalp EEG dataset from Physionet (https://physionet.org/) was first introduced by Shoeb *et al*. [24]. It is one of the scalp EEG datasets frequently used for seizure detection or prediction investigations [48,71,72]. The data are obtained from 23 paediatric patients or 24 subjects (since subject 21 was obtained 1.5 years after subject 01 from the same patient). The male patients are in the age group of 3 to 22 years, whereas female patients are in the age group of 1.5 to 19 years. A minimum of two types of seizures were recorded in each of the subjects among a total of three different categories, including partial (SP), complex partial (CP) and generalized tonic-clonic (GTC) seizures. Seizures in the hippocampus (in the deep temporal) area were recorded for eight subjects, and another 11 had seizures in the neocortical brain location. A total of 24 subjects contain 686 records with more than 950 hours (h) of recordings. The recording was done continuously using 256 Hz, 16-bit resolution for 1–4 h for each record. A total of 22–36 EEG channels were used to record the data from the subjects. However, in many cases, the record contains other signals along with the EEG signal, such as ECG, vagal nerve stimulus (VNS) and other dummy signals. Of all records, 141 have at least one seizure event (called seizure records), totalling 198 seizure events of lengths starting from 7 to 753 second (s). There are a total of 545 normal recordings (non-seizure records) for all the subjects, with no seizure events at all. The dataset has an approximate total of 11 693 s and 3 417 165 s of seizure and non-seizure events, respectively.

The EEG signals were recorded using the different combinations of electrodes (or channels), which can be termed channel groups. Five different channel groups were found in this dataset, each with 22–36 unique EEG channels. The electronic supplementary material presents more details about the channel in table 1 of electronic supplementary material, appendix C. One of the five groups is considered for analysis in this study. Although we did not intend to select channels for this study, we chose this particular group because it was used for most of the records in the dataset. This particular channel group has been used by 655 records (out of 686), which contain 175 seizure events (out of 198). There are 22 unique channels in this selected group and those are: FP1-F7, F7-T7, T7-P7, P7-O1, FP1-F3, F3-C3, C3-P3, P3-O1, FP2-F4, F4-C4, C4-P4, P4-O2, FP2-F8, F8-T8, T8-P8, P8-O2, FZ-CZ, CZ-PZ, P7-T7, T7-FT9, FT9-FT10 and FT10-T8. Signals from each of the 22 individual channels were segmented with a 5 s non-overlapping window. A total of 1985 seizure segments with 5 s duration and 6 83 433 non-seizure segments were obtained from the CHB-MIT dataset. A brief summary of the data used in this study is presented in table 1. However, a more detailed version of the data description can be found in table 2 of appendix C in the electronic supplementary material.

**Table 1.** Summary of total available data in the dataset and the portion of data that is considered and observed for this study. Due to the selection of channel groups to make the experiments consistent with all the subjects, the total amount of seizure and non-seizure-related data is reduced. The column on the right-hand side represents the amount of data that is observed for this study.

criteria	total available	observed
subjects	24	24
records	686	655
seizure records	141	136
seizures events	198	175
signal frequency	256 Hz	256 Hz
channel groups	5	1
channels	18–32	22
signal types	EEG, ECG, VNS, –	EEG
seizure event samples	11 693 s	9925 s
seizure to non-seizure ratio	—	1:344
data balancing	—	imbalanced test
extracted features	—	92 (per channel)

*Notes:* The dash (—) represents that the data are unavailable or not represented.

This dataset has a higher class imbalance as the non-seizure segments are more than 344 times larger in number than the seizure segments. The data were kept imbalanced to observe the real-world seizure detection scenario from continuous EEG, as in general, data will have more non-seizure duration than the seizure event duration. The signals from the dataset were used without any changes in the properties of the signal. No other preprocessing (e.g. filtering, artefact removal, etc.) was applied to the signal, as these may alter its original characteristics, as previously described. This alteration also may lead to changes in the actual properties of the seizure portion of the signal.

### 2.3. Segmentation and feature extraction

This study used a 5 s non-overlapping window to segment the signal from the channels. The reason for choosing a 5 s window is that segments that are too small or too large might lead to more inaccurate classification. Too small segments might not have enough effective patterns that the extracted feature can capture, while too long segments might dilute the actual pattern of the data. In addition, several studies have achieved good results with this length [60,73,74]. Although the effect of different segment lengths could be observed, that is kept out of the scope of this study as future work.

Different linear and nonlinear features extracted from the EEG signal have been found to be useful for ES detection [75–79]. Thus, from each 5 s scalp EEG segment, different linear and nonlinear features from the time and frequency domains were extracted, which are listed in table 3 of appendix C in the electronic supplementary material. Entropy profiling is one of the important features that can describe heart-rate variability very well [80]. Some secondary features from the sample entropy profile were used in this study as the time-domain nonlinear features. In addition, several statistical features from five commonly used frequency sub-bands of EEG (alpha, beta, delta, theta and gamma) were extracted. Similarly, spectral power-based nonlinear features were extracted from the raw EEG signal. All features used in this study are listed in table 3 of appendix C, and their details are provided in appendix A of the electronic supplementary material.

In summary, 92 features were extracted from each 5 s scalp EEG segment. These features were extracted using a library developed by our team that is available on our GitHub repository.^1^ All these hand-crafted features were then stored along with the original label (if the segment is seizure or non-seizure) for each segment. When preparing the dataset, seizure segments were labelled as 1, and non-seizure segments were labelled as 0. Some segments contained a portion of both the seizure and non-seizure EEG. The labels for those segments were decided as seizures if they contained 60% (3 s in our case) or more seizure portions, and non-seizure otherwise.

### 2.4. Feature selection

Since this is a multi-channel study and 92 features were extracted for each channel, it resulted in a total of (92 features × 22 channels) = 2024 features for each epoch/segment, which are huge in number and might cause the curse of dimensionality. Thus, feature selection was used to remove the least effective features. The feature selection was done on sample data using four different feature selection methods, namely, chi-squared, correlation (with target), decision tree (DT) classification importance, and RF (permutation importance-based). The sample data comprised the segments taken from one channel of randomly selected five subjects. The feature selection process was repeated for randomly chosen five different channels, and each feature’s average importance score was calculated at the end. To select features, the cumulative average importance scores (on a scale of 1.0) of all feature selection methods were compared as shown in electronic supplementary material, figure 2 where a threshold of 0.9 was used (empirically) as a cut-off point. The minimum number of features required to achieve 0.9 cumulative score by the four feature selection methods is listed in table 4 of appendix C in the electronic supplementary material. Based on the cumulative average importance scores and the minimum number of features that achieved the cut-off point, the chi-squared feature selection method was chosen for this study. Thus, 34 features out of 92 were selected for each channel, and those are listed in table 5 of appendix C in the electronic supplementary material. Finally, from 22 channels, a total of (34 features × 22 channels) = 748 features were then used for the classification of seizures.

**Figure 2.**
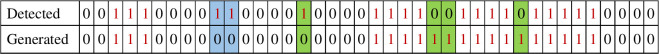
The process of event generation from the results from all the segments. The detected portion is the actual segment detection by the ML model and the generated one is the new/modified results after applying post-processing for the corresponding segments. After combining the results of the segments sequentially, the labels of the seizure segments (with 1) in a continuous non-seizure event are replaced with non-seizure labels if the duration is 10 (ten) s or less (as the first two changes indicated using the shaded areas). Similarly, the labels for non-seizure segments (with 0) in a continuous seizure event are replaced with seizure labels if the duration is 10 (ten) s or less (as the last two changes indicated using the shaded areas).

### 2.5. Data splitting and training data balancing

Data splitting was done based on the demand of the experiments used in this study. Two sets of cross-subject experiments were conducted in this study to observe the event detection performances. One used SW 5-fold cross-validation, and the other used the LOO cross-validation mechanism (random samples (RS) are not distributed in different folds). All 24 subjects were randomly grouped into five groups for the first case. The grouping was done this way so that each group has some random subjects that are unavailable in any of the other four groups, similar to other previous studies [51,60]. So, the classifier model was trained using only four groups and tested with the left-out group for seizure segment detection. For all five groups, each was tested based on the training using the remaining four groups of subjects. For the LOO mechanism, similar to some related studies, each of the 24 subjects was considered the test set, and the model was trained on the rest of the 23 subjects other than the only test subject in a repeated fashion [41,51,81]. Both mechanisms ensured the training-test process, in which the model was not tested with the data of any subjects it was trained on.

Once the training-test splitting was done, we applied class balancing on the training dataset. As described earlier about the imbalance of classes in the CHB-MIT dataset, the total duration of the non-seizure portion is higher than the seizure portion. If we keep the training set highly imbalanced, the model will most likely be biased towards the majority class (non-seizure). For this reason, the model was trained on the balanced data using the under-sampling mechanism, but the test data was kept as it was. We have used under-sampling since over-sampling or augmentation may lead to generating seizure patterns that are not actual seizures.

### 2.6. Classification

RF classifier is an ensemble ML classifier that achieved greater performance scores compared with other ML methods on average as found in previous studies [47,60,62,63]. The ML models are more explainable compared with the DL models; for that reason, the ML model, specifically the RF model, was chosen as an initial model for this study. Investigating the performance of other similar methods would be very interesting to observe and would be a good finding itself. To focus on the main goal of this study, this criterion is left out for future work. However, different ML models were observed (using PyCaret Python library^2^) on the sample dataset explained in §2.4 to select the final model for this experiment. Table 6 of appendix C in the electronic supplementary material lists the performance scores of all the models for that observation. Even though other models were not tested in this study, the overall performance scores for the sample data shown in this table helped with model selection. Based on these performance scores, the RF model was chosen for this study over other ML models. The RF classifier generated labels (predicted on test data) for each segment for the corresponding features were later used for event analysis.

The RF classifier can be equipped and tuned with many different parameters to perfect the classifier so that we can find a more robust and generalized model. Of all the possible parameters that can be used, in this study, the ‘ n_estimators ’ parameter was used and tuned for the model. The ‘ n_estimators ’ is the number of trees that can be used to actively participate in the selection criteria of the voting score of RF, or simply the number of trees used in the forest. The model was checked and tuned for the ‘ n_estimators ’ parameter with the values—15, 21, 30, 50 and 75. To optimize these parameters using Grid-search and choose the best model with optimized parameters, a subject-level random 30% of the training data was used for validation purposes.

### 2.7. Post-processing

As obvious, every subject contains many long-term records, and all the records for the same subject can be considered continuous (as the dataset describes). For event detection, all the records from the same subject are sorted based on their temporal order. This way, a large chunk of a logical unit of signal from every single subject is achieved and used for event observation. The segment-based result was kept as it is, based on the counts of the segments that were properly classified. Unlike seizure segment detection, simply counting the number of segments is not enough for performance evaluation for event detection. For event detection, checking the continuity of the detected segments, along with the start and end of the event, is also important. Since the goal of this study is to observe the performance of event detection, the performance was measured after some post-processing for event evaluation. However, some segment-based performances are also presented in this study. Due to the segmentation process, individual seizure event was distributed to several segments. For event detection, the segment-based results of the models were combined sequentially in the same order as the original data to generate a combined result, similar to some previous studies [63]. For event calculation, the seizure events that are less than or equal to 10 s in length in the combined result were ignored (and replaced with 0). Seizures can also be detected from shorter segments such as 2 or 5 s segments [41,60,82]. However, for better seizure detection, a 10 s latency has been found to be comparatively more effective in many previous studies [18,83]. For that reason, the segment length chosen for our study is 5 s as described in earlier section as well. Thus, to make an event, at least two such segments with a total length of 10 s are selected as the threshold for post-processing. Any non-seizure event between two seizure events was ignored (and replaced with 1), and those two events were considered continuous when the gap between them was less than or equal to 10 s. The event generation process from the segment results is shown in figure 2. The original model-generated example output (labelled as *Detected* in the figure) contains seven discrete events, but after post-processing (the changes marked with blue shadow) the three actual events are found (2nd row labelled as *Generated*). Then, the post-processed (generated) seizure labels were compared with the true target labels.

To compare, if any detected event (model result) covers 70% of the duration of the corresponding original seizure event (true data label), then it is considered that the model detected that event correctly. If there is one long true event (seizure sequence) and the model detects more than one small event within that period, then only one of the events is counted (preferably the largest one). This is done to ensure that there is no more than the actual number of events detected by the model and increases the sensitivity (*sen*) score incorrectly. On the other hand, the events detected in the non-seizure zones were kept as they were so that the actual misclassification was counted. After post-processing, different performance metrics were used to measure the performance of event-based seizure detection. In brief, a seizure event is considered to be detected if the original data contains an event (stream of continuous seizure segments) and the model detects at least 75% of that event duration after post-processing. On the other hand, a false detection is considered whenever the model detects an event (stream of continuous seizure segments) as a seizure, but that portion was non-seizure in the original data.

**Figure 3.**
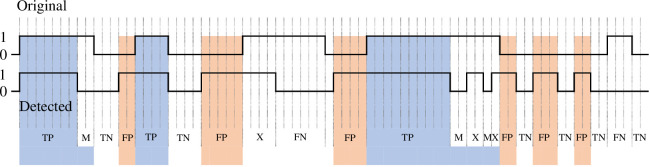
The process of event matching in post-processing by emphasizing event detection and missing events or unnecessary event detection. The digital waveform at the top represents the original events with 1 as seizure and 0 as non-seizure. Similarly, the waveform at the bottom represents the detected events by the segment-based systems (after event generation process) with a similar annotation for seizure and non-seizure events. The shaded green region shows the correct event detection or TP and the shaded orange region represents the false detection or FP. For the region, M , the FN is disregarded since the TP of both sides covers at least 70% (the event threshold) of the original seizure event. On the other hand, the region X is not considered TP since the total detected seizure duration does not cover the event threshold in the original event.

The event matching criteria with different situations are presented in figure 3. Here the *Original* label indicates the true seizure labels from the dataset and *Detected* refers to the *Generated* labels from the previous step. Small mistakes (false detection) are closely observed and punished to make the seizure detector more accurate and realistic. On the other hand, less flexibility is given to the detection of the correct events, which are bound to be at least a 70. This mechanism is similar to the overlap (OVLP) method explained in the previous study but not the same [64,84,85]. However, instead of the event-matching principle of ‘any’ part of the original event being detected by the ML model, we followed this 70% overlap principle to make the system more reliable. From figure 3, if the detected segments cover at least 70% of the original event, it is considered true positive (TP). The regions marked with *M* are discarded because of the above condition, even though they are false negatives (FN*s*). The regions marked with X are also discarded, even though they are partially TP*s*. On the other hand, if the system detects a seizure but there is no seizure in the original signal, it is considered a false positive (FP). For example, in this figure, let us consider it is for a 2-h-long data. Since the system detected 3 out of 5 seizure events correctly, thus the (sen) will be 60%. On the other hand, it misclassified the portion of the seizure event six times in 2 h; thus, the false detection rate (FDR) is 3/h.

### 2.8. Performance metrics

The performance of the study was measured using the following metrics. For ES analysis, the seizure event is considered a positive (1), and the non-seizure is negative (0).

— Sensitivity: The TP rate corresponds to the proportion of positive data points that are correctly considered positive with respect to all the positive data points. This is usually called Sen or rec (rec).

$$Sen\;=\;\frac{TP}{TP+FN}$$

— False detection rate: The FDR is the hourly rate of misclassification of seizure event. It is the measure of the total misclassified seizure events divided by the total duration in hours.

$$FDR\;=\;\frac{FP}{total\_hours\_observed}$$

where,

— *TP*: The cases in which the model predicted positive and the actual output was also positive.
— *True negative (TN)*: The cases in which the model predicted negative and the actual output was negative.
— *FP*: The cases in which the model predicted positive and the actual output was negative.
— *FN*: The cases in which the model predicted negative and the actual output was positive.

Since the event-based analysis differs from segment detection and there is a high imbalance in the data classes, traditional metrics used for segment detection are inappropriate for this case. The more practical way to evaluate event-based performance is to use the Sen and FDR. These two metrics provide information on the number of seizure events that are correctly classified and the number that are misclassified per hour. In this case, the Sen score reports the seizure event detection performance, and the FDR score informs the efficacy of handling highly imbalanced non-seizure events. It is to be noted that the performances were calculated based on event count. The key information here is how many original events were properly detected and how many were missed.

## 3. Results

**Figure 4.**
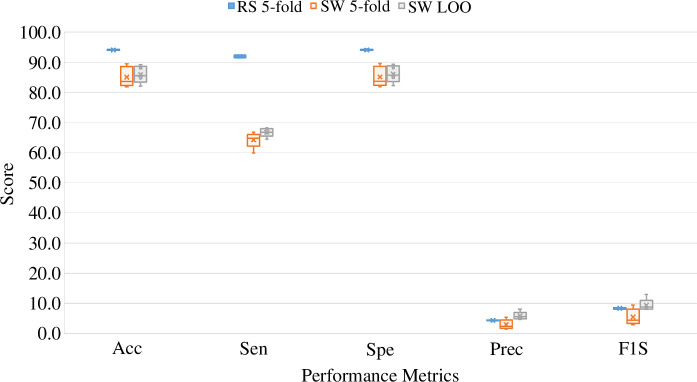
The segment-based scores in terms of Sen scores for RS 5-fold, SW 5-fold and SW LOO analyses. The Sen score is presented as a percentage result. This box plot shows the Sen scores on average with the distribution of the results for five runs for the analyses.

**Figure 5.**
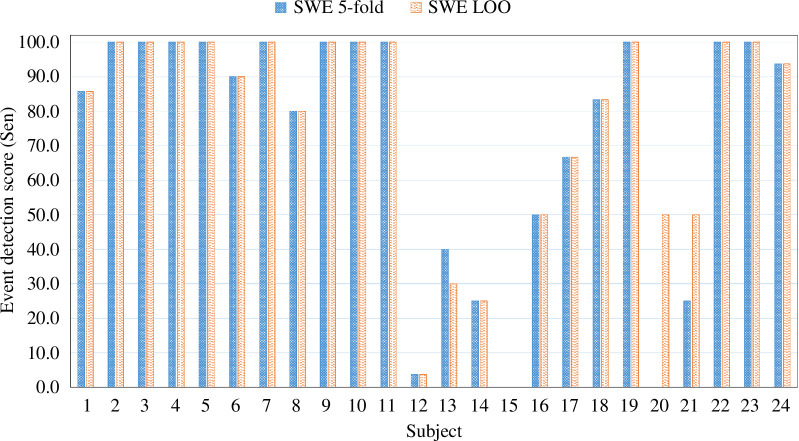
The event-based scores in terms of Sen scores for the SW cross-subject event detection (SWE) 5-fold and SWE LOO analyses for the individual subjects. The Sen score is presented as a percentage result.

This section presents both the segment-based and event-based results. The first part of this section will discuss the segment-based results. The event-based results derived from the segment-based results will be discussed in detail in the later part. Keeping event detection in mind as our main target, segment detection performances were also observed. Seizure segment detection was done using different approaches: RS 5-fold, SW (cross-subject) 5-fold and SW LOO. In the first approach, the training and test samples were arbitrarily taken from different subjects for an 80:20 ratio for training and testing. On the other hand, for the latter two approaches, training and test data were divided SW as explained in §2.5. The experiment was run five times for all these approaches to get segment-based results. The segment-based comparative results for these three approaches are presented in figure 4. However, table 7 of appendix C in the electronic supplementary material shows more comprehensive detailed results for RS 5-fold. The graph of the segment-based results clearly shows that the RS 5-fold results show consistency across the runs, whereas the other two approaches have slight variations. For accuracy (Acc), Sen and specificity (Spe) scores, SW experiments performed significantly less than the RS 5-fold. On the other hand, a slightly different result has been found for SW LOO results. An increase in precision (Prec) has been found in the SW LOO approach, resulting in an improved *F1S*. This exception is expected to occur because comparatively large data (23 out of 24 subjects) have been used for training. This may also happen because of largely imbalanced classes in the test data. However, according to some earlier studies, *F1S* is not always a great choice for performance measurement when there is a higher imbalance in class data [86]. Thus, we stick to the two performance metrics defined in the earlier §2.8. These are the most common metrics used in earlier studies; thus, they can be used to compare our study with those. Since the variation in experimental results (of 5 runs) for both SW approaches is insignificant, we have taken one experimental result from each of these two SW approaches for event-based analysis. As explained in the earlier section, two types of cross-subject analytical approaches were observed for event detection in this study. The first is the 5-fold cross-subject analysis, and the other is the SW LOO cross-subject analysis. For each analysis, the observation was done from an event perspective, and the performance score was presented for the individual subjects. The event-based performance scores in terms of Sen and FDR are presented in figure 5. The corresponding detailed numeric results are tabulated in table 2. The SW 5-fold and LOO results were shown in both of the presentations. As can be observed, both of the analyses exhibit almost similar results for each subject. Events were appropriately detected similarly, indicating that the data leakage was prevented well for both of these analyses. Both the SW 5-fold and LOO analyses detected the seizure events in a similar fashion as described below. For 18 subjects, both analyses produced the same Sen scores with slightly different FDR scores. Both achieved 100% Sen scores for 11 out of 24 subjects. For the other 5 subjects, both cross-subject analyses achieved more than 80% of the Sen score. The SW LOO testing produced comparatively better output for all subjects except for subjects 13 and 21 than the 5-fold analysis.

The 5-fold analysis failed to detect seizure events for subject 21. However, both analyses failed to detect any seizure event for subject 15. A similar scenario is found for both analyses in which only one seizure event was properly detected for subject 12 with a lower Sen score. The exact seizure and non-seizure counts in event-based analysis with the corresponding detection results are also presented in table 2.

**Table 2.** The event count and performance score in terms of Sen and FDR of event-based investigation for SW both 5-fold and LOO analyses. Event count is presented with respect to the total number of actual seizures and non-seizures. The Sz indicates the seizure count and Nsz is the non-seizure count for the corresponding investigation. The performance scores of event-based investigation for both 5-fold and LOO analyses based on SW data splitting. The performance scores are presented with the Sen score on a scale of percentages and FDR score on a scale of missed event counts per hour. Rows with increasing intensities of olive colour indicate the lower performances for the different subjects (for the corresponding experiments). The overall values for the event count are calculated by adding the event values, and the performance scores are calculated by averaging the score values.

	event count						performance score			
	total		5-fold		LOO		5-fold		LOO	
subj	Sz	Nsz	TP	TN	TP	TN	Sen (%)	FDR (h)	Sen (%)	FDR (/h)
1	7	8	6	5	6	4	85.71	0.37	85.71	1.06
2	3	4	3	1	3	1	100.00	1.56	100.00	2.70
3	7	8	7	2	7	4	100.00	0.61	100.00	1.10
4	4	5	4	1	4	1	100.00	2.55	100.00	3.97
5	5	6	5	0	5	0	100.00	14.56	100.00	15.03
6	10	11	9	1	9	0	90.00	14.82	90.00	14.15
7	3	4	3	0	3	0	100.00	2.64	100.00	2.36
8	5	6	4	1	4	1	80.00	16.10	80.00	10.80
9	4	5	4	0	4	0	100.00	11.30	100.00	12.51
10	7	8	7	0	7	0	100.00	9.12	100.00	8.20
11	3	4	3	1	3	1	100.00	1.15	100.00	1.10
12	27	28	1	23	1	20	3.70	2.80	3.70	3.63
13	10	11	4	0	3	2	40.00	15.27	30.00	9.82
14	8	9	2	0	2	0	25.00	12.81	25.00	3.81
15	20	21	0	16	0	21	0.00	0.59	0.00	0.00
16	2	3	1	0	1	0	50.00	9.89	50.00	10.10
17	3	4	2	2	2	2	66.67	1.90	66.67	2.20
18	6	7	5	5	5	5	83.33	0.20	83.33	0.38
19	3	4	3	0	3	1	100.00	0.52	100.00	0.31
20	8	9	0	7	4	6	0.00	2.61	50.00	3.26
21	4	5	1	3	2	2	25.00	0.12	50.00	0.34
22	3	4	3	0	3	0	100.00	1.32	100.00	2.84
23	7	8	7	3	7	3	100.00	0.49	100.00	0.38
24	16	17	15	8	15	6	93.75	4.32	93.75	4.93
overall	175	199	99	79	103	80	72.63	5.32	75.34	4.79

With these per-subject results, this study achieved the average Sen scores of 72.63% and 75.34% for SWE 5-fold and LOO analyses, respectively. The standard deviations of the results are 36.58 and 32.86 for the Sen score for the respective analyses. Average FDR scores of 5.32/h and 4.79/h were generated for SWE 5-fold and LOO analyses, respectively. Subject numbers 5, 6, 8, 9, 13, 14 and 16 produced higher FDR scores in both analyses. The best performance was reported for subject 23 with 100% Sen and the lowest FDR score of 0.49/h for the 5-fold analysis. Conversely, the best performance for SWE analysis was for subject 19, with 100% Sen and an FDR score of 0.31/h. Overall, the results indicate that the Sen scores are comparatively lower for subjects 12–15, and the model also struggled with subjects 20 and 21. Other than this, the result shows a moderately fair overall performance for this type of experimental setting that considers all the critical parameters or factors.

## 4. Discussion

The results show that the proposed method is capable of detecting ES with great efficiency considering the real-world situation. The results have shown the perfect Sen scores for most subjects except for a few (2–7) subjects out of 24. A tremendous performance improvement was observed in some studies if these subjects were excluded [44,45,47]. It is very challenging to achieve outstanding performance in this study setting for the following reasons, which coincide with the main contributions of this study:

— The dataset is highly imbalanced in terms of classes, and all data were considered for testing. This is related to the large data imbalance and entire data consideration. This also reflects the scenario of the continuous EEG data. (Contribution 2)
— The higher inter-subject variability of the signal is there in the EEG signal. This is linked to the subjective data leakage problem and the need for cross-subject validation. (Contribution 3)
— The event-based investigation has to deal with continuous segment detection, and there are fewer seizure events in long continuous scalp EEG. This is coupled with real event detection rather than segments. (Contribution 4)

Since these problems are directly associated with this study’s contributions and important factors, it is better to discuss the results from these perspectives. Most DL methods can usually achieve better performance; however, the key insights of the seizure detection process remain a mystery [37,42,82]. In ML-based classification tasks, the test data is usually expected to be unrevealed to the model during training. However, the test data is expected to have some patterns similar to the training data. The highly fluctuating subjective results (Sen and FDR scores) echo the concept of inter-subject variability of the EEG signal, as proven by previous studies. However, the method applied in this study captures most of the variations of the seizure signals. Thus, near-perfect Sen scores have been achieved for most of the subjects. The less-performing subjects might have some rare seizure patterns that were never encountered during training using other subjects. These subjects need further intensive investigation in the future. Since this study perfectly tackles the subjective data leakage problem, the model learns no rare patterns for a single and specific subject. Another notable factor is that there is not only data imbalance in this dataset but also seizure event imbalance across subjects. Some subjects have fewer seizures compared with other subjects. Conventionally, when these subjects are in the training set, the model has less opportunity to learn the seizure pattern, which leads to lower Sen scores and more misclassification.

On the other hand, the higher FDR rate is due to such a high imbalance in data because the model has limited accurate knowledge about the pattern of seizure and non-seizure events. In addition, there are very long-duration non-seizure portions in the test sets, all the patterns of which never appeared in the training set due to data balancing in training sets. The model could not learn these completely unknown patterns. Hence, the model became confused and misclassified more, resulting in a large number of FDR scores. This type of phenomenon was also observed in another study for the CHB-MIT dataset [87]. Another factor is that during post-processing of the data for event detection, the minimum gaps between two seizures or two non-seizures were kept to 10 s, as this was found to be the standard length in previous studies. The model might have detected some short-duration seizure events apart from each other for long-duration non-seizure portions and was discarded in the post-processing phase. In the same single non-seizure portion, the post-processing method might have detected two or more false seizures for some short duration in many situations. All these reasons can also largely increase the FDR.

Some recent and highly related studies are available for seizure detection based on the CHB-MIT dataset. Those studies are compared with the proposed study, and the comparison is presented in table 3. In this part, we only focus on how efficiently the models can detect seizure events and how often they miss the original events. It is not possible to compare this study directly with previous studies due to the differences in the experimental parameter choice. The parameters include data, the nature of the analysis, the target of the research and other criteria, which are directly related to the factors we discussed in §1. The comparison table lists all the studies that considered those different factors, criteria or parameters for analysis. The studies in the table are listed based on feature explainability, whether the complete dataset was tested, whether the cross-subject versus fold-based analysis or subject-specific analysis was done, and whether event-based or segment-based analysis was undertaken.

**Table 3.** Summary of the performance scores and the factors considered during ES detection on CHB-MIT dataset. This table includes different ML and DL methods individually or combined with other methods such as preprocessing and post-processing. In this comparison of the entire ES detection process, some criteria are considered, such as the explainability of features, changes in data (considered fewer data, under-sampling, training over-sampling, channel alteration, filtering, etc.), cross-subject testing (to avoid data leakage) and event (or onset or segment) detection. Finally, the performance scores are presented in the last columns in terms of Sen and FDR where applicable. The tick mark represents that the criterion is applied/available, and the cross mark represents that the criterion is not applied or available. NR represents that the corresponding data are not reported. The numbers associated with the column headings indicate the corresponding contribution that this study covers.

					performance scores	
study	feature explainability (1)	entire dataset (2)	cross-subject analysis (3)	event-based detection (4)	Sen *(*%)	FDR (/h)
Abdelhameed *et al*. [82]	✗	✗	✗	✗	98.72	✗
Gao *et al*. [37]	✗	✗	✗	✗	98.72	✗
Amiri *et al*. [88]	✓	✗	✗	✗	98.44	✗
Sun *et al*. [45]	✓	✗	✗	✗	96.79	✗
Shen *et al*. [44]	✓	✗	✗	✗	96.15	✗
Jiang *et al*. [68]	✓	✗	✗	✗	98.71	✗
Zeng *et al*. [63]	✓	✗	✗	✗	96.98	✗
Bhattacharyya *et al*. [47]	✓	✗	✗	✗	97.1	✗
Wang *et al*. [42]	✗	✗	✗	✓	99.31	0.2
Li *et al*. [43]	✓	✗	✗	✓	98.47	0.63
Vidyaratne *et al*. [18]	✓	✗	✗	✓	97.00	0.10
Boonyakitanont *et al*. [62]	✓	NR	✗	✓	76.54	0.09
Pale *et al*. [64]	✓	NR	✗	✓	34.10	0.05
Raghu *et al*. [40]	✓	NR	✓	NR	97.28	0.57
Wei *et al*. [41]	✗	✗	✓	✓	90.57	✗
Hossain *et al*. [51]	✗	✗	✓	✗	90.00	✗
Thodorof *et al*. (2016) [81]	✗	✗	✓	✗	85.00	0.80
Zhou *et al*. [59]	✓	✗	✓	✗	84.67	✗
Wu *et al*. [60]	✓	✗	✓	✗	82.98	0.57
Zhao *et al*. [58]	✗	✗	✓	✗	77.42	✗
Wei *et al*. [41]	✗	✗	✓	✗	72.11	✗
Jana *et al*. [57]	✗	✗	✓	NR	55.63	✗
proposed study (SWE 5-fold)	✓	✓	✓	✓	72.63	5.32
proposed study (SWE LOO)	✓	✓	✓	✓	75.34	4.79

The comparison table shows that only a few of them investigated the event-based seizure classification or seizure onset detection [17,18,41,42,62,64]. Some event detection-based studies suffered greatly from achieving higher performance scores, especially the Sen scores [62,64]. It is clear from the table that the overall performances of the models that did cross-subject analysis are not very high compared with the other studies that had considered different analysis approaches [41,57–59,81]. Significant performance degradation can be noticed in cross-subject analysis in many studies [41,57,58]. A performance drop is expected when considering both criteria (cross-subject and event-based) in analysis. Only one study was found in the literature that dealt with both of these issues [41].

Additionally, more criteria for considering the robustness of the model (using the entire dataset) put the model under more challenges, and the model struggles significantly to score better. The data imbalance problem, which reflects the real-world scenario of continuous EEG data, pushes the model into harder situations, where it faces unknown patterns not learned during its training phase. This is one of the main reasons why many studies have not explored this side. To test the model’s performance, almost all the studies considered part of the data. The data selection for the studies was done using one of several methods, such as partial subject selection, considering only seizure records, filtering, under-sampling, modification or reduction in data channels, etc.

The results of this study and the summary of the literature (table 3) show it is more challenging to implement an ES detection system in a real-world scenario if all the factors are considered. The segment-based results shown in §3 also align with table 3. They show the difficulty of achieving better performances (Sen) when the cross-subject analysis is undertaken for a generalized seizure detection system. The summary table also shows the trend of significant performance reduction when event detection is done. Combining these criteria (cross-subject analysis and event detection) with considering the entire dataset for testing adds additional complexity to the ML model. Thus, when adding more real-world factors to the models, the ML models generally tend to achieve relatively less seizure detection performance (Sen). This finding opens up a broader scope of exploration for other researchers to tackle these challenges and develop a more realistic solution. However, proper modelling of the problem might help researchers develop a better-performing and generalized solution.

## 5. Limitations and future direction

This study focuses mainly on different real-world factors that need to be reflected in the analysis based on recent related studies. The analytical implementation was simplified only to observe the impact of those factors. However, there is scope to extend the observation further to investigate the effects of different parameters. Various preprocessing methods and window lengths can be observed for seizure detection using a similar setup. Different features might be able to capture more meaningful information from different data lengths of the original signal, and extracting features from fixed-length data can sometimes be less efficient. Thus, independent observation of feature extraction with variable data length can be investigated in seizure detection. In-depth statistical analysis to reveal the underlying relationship can also be useful in seizure detection. Other different ML models can also be compared with test the generalizability of a model for this experimental setting. Different hand-crafted features, especially those from the wavelet domain, can also be applied and tested to investigate their efficacy in seizure event detection. On the other hand, the DL models can also be observed for this purpose if the feature explainability is not essential. The post-processing method can also be improved by implementing different thresholds for event gaps and various techniques for seizure event estimation. Additional knowledge from post-processing might help produce other fundamental findings that could reduce dependence on post-processing in the future. Since this is a multi-channel analysis, it would be interesting to observe the same for individual channels or some selected channels. Since evenly balanced data was used to train the model, that might not be enough to learn more different non-seizure patterns. It would be great to observe the same result for slightly imbalanced data. This experiment is limited to CHB-MIT dataset; observing the event detection efficacy across the different datasets would be great. This study was limited to a specific scope for simplicity and focused only on the criteria defined in §1. Some of these unexplored areas are our target to observe in the near future.

## 6. Conclusion

In this study, we investigated the effect of different important analytical factors altogether in ES detection using the CHB-MIT dataset. The factors include (i) considering the entire dataset for testing; (ii) subjective data leakage prevention using cross-subject and developing a generalized model; (iii) detecting ES events; and (iv) using explainable features. To the best of our knowledge, this study considers all these four crucial factors for the first time in ES detection. A total of 92 hand-crafted features from 22 scalp EEG channels were extracted. Using chi-squared, 32 selected features from each channel were used to detect seizure events by the RF model. The analyses are categorized mainly into segment- and event-based observations. Segment-based results show that subjective variation and similarity play a vital role in seizure detection. Hence, the RS 5-fold approach achieved a Sen score of 91.88% for seizure detection, which is significantly higher than the cross-subject performances. On the other hand, the other two SW cross-subject analysis approaches, namely, 5-fold and LOO observations, were able to achieve 64.24 and 66.75%, respectively. Cross-subject segment-based results were used to calculate event-based results using a post-processing method. For the event-based analyses, the cross-subject 5-fold and LOO approaches performed similarly with the Sen scores of 72.63 and 75.34%, respectively. Only a few subjects were found for which the model performed less efficiently, leading to the FDR of 5.32/h and 4.79/h for the respective analysis. Both cross-subject experiments (5-fold and LOO) perform very similarly, with the LOO obtaining a little better performance because of the model trained on more data.

Considering all the factors mentioned above that simulate a real-world environment, the overall performance obtained in this study is more realistic and fair. The experimental results and the trend of the literature show that developing a generalized seizure event detection system with the above-mentioned necessary criteria is more challenging. The findings of this study may help guide real-world application-oriented research a step further that can be used to develop devices for actual clinical applications.

## Data Availability

Used data are publicly available on Physionet: https://doi.org/10.13026/C2K01R [89,90], under license: Open Data Commons Attribution License v1.0: https://www.physionet.org/content/chbmit/view-license/1.0.0/. According to the License terms in section 3.0 'Rights granted' subsections—a, b, d and e; the following rights are granted to the users: Extraction and re-utilization; creation of derivative databases; creation of temporary or permanent reproductions; distribution, communication, display, lending, making available or performance to the public by any means and in any form, in whole or in part. Electronic supplementary material is available online at [91].

## Footnotes

^1^
https://github.com/WWM-EMRAN/DIHC_FeatureManager

^2^
https://github.com/pycaret/pycaret
